# 胸腔镜解剖性肺段切除术技术要点

**DOI:** 10.3779/j.issn.1009-3419.2016.06.16

**Published:** 2016-06-20

**Authors:** 亮 陈, 卫兵 吴

**Affiliations:** 210029 南京，南京医科大学第一附属医院胸外科 Department of Thoracic Surgery, the First Affiliated Hospital of Nanjing Medical University, Nanjing 210029, China

**Keywords:** 胸腔镜, 肺段切除术, 3D-CT, 支气管及血管造影, Thoracoscopy, Pulmonary segmentectomy, Three-dimensional computed tomography, Bronchography and angiography

## Abstract

由于肺段支气管、血管的解剖变异繁多，胸腔镜肺段切除术的技术较肺叶切除术更加精细复杂。术前三维CT支气管血管成像（three dimensional-computed tomography bronchography and angiography, 3D-CTBA）可以显示肺段的解剖结构以及肺段支气管、血管的变异，明确肺部结节的肺段归属，有助于制定手术计划。术前结节定位在胸腔镜肺段切除术中起着重要作用。解剖性肺段切除术的技术包括：分别切断靶段动脉、支气管、段内静脉，保留段间静脉，采用膨胀萎陷法确定肺段间交界面，使用电刀和/或切割缝合器分离段间肺组织。恶性结节行肺段切除术时必须确保切缘宽度≥2 cm，或≥肿瘤直径，同时需要进行N1、N2站淋巴结采样及冰冻切片分析。胸腔镜解剖性肺段切除术的并发症发生率较低。肺段与肺叶的解剖关系为，若干个呈不规则锥形结构的肺段组成一个肺叶，段间静脉行走于肺段间。本中心探索出一种按照锥形结构原理自肺叶中分离出肺段的方法，并命名为“锥式肺段切除术”。“锥式肺段切除术”的技术涵盖了准确判断和处理靶段支气管和血管以及解剖性分离段间交界面，可以实现精准的肺段切除。

## Abstract (en)

Thoracoscopic segmentectomy is technically much more meticulous than lobectomy, due to the complicated anotomical variations of segmental bronchi and vessels. Preoperative three-dimensional computed tomography bronchography and angiography, 3D-CTBA) could reveal the anatomical structures and variations of the segmental bronchi/vessels and locate the pulmonary nodules, which is helpful for surgery planning. Preoperative nodule localization is of vital importance for thoracoscopic segmentectomy. Techniques involved in this procedure include dissection of the targeted arteries, bronchus and intra-segmental veins, retention of the inter-segmental veins, identification of the inter-segmental boarder with the inflation-deflation method and seperation of intra-segmental pulmonary tissues by electrotome and/or endoscopic staplers. The incision margin for malignant nodules should be at least 2 cm or the diameter of the tumor. Meanwhile, sampling of N1 and N2 station lymph nodes and intraoperative frozen section is also necessary. The complication rate of thoracoscopic segmentectomy is comparatively low. The anatomic relationship between pulmonary segments and lobes is that a lobe consists of several irregular cone-shaped segments with the inter-segmental veins lies between the segments. Our center has explored a method to separate pulmonary segments from the lobe on the basis of cone-shaped principle, and we named it "Cone-shaped Segmentectomy". This technique could precisely decide and dissect the targeted bronchi and vessels, and anatomically separate the inter-segmental boarder, which ultimately achieve a completely anatomical segmentectomy.

手术是早期肺癌的首选治疗，微创、解剖性切除和节约肺组织的手术方式有利于提高早期疗效而不影响肿瘤学疗效^[1]^。虽然肺叶切除术目前仍是早期肺癌的标准术式，但近年多中心回顾性研究发现肺段切除术治疗Ⅰa（T1aN0M0）期肺癌的疗效和肺叶切除术类似，复发率和5年生存率无差异^[2-4]^，并可减少并发症和死亡率，有利于保护术后肺功能^[5, 6]^。胸腔镜解剖性肺段切除技术也在不断探索中进步。由于肺段解剖复杂，变异较多，要达到胸腔镜下完全解剖性肺段切除术难度较大，肺段切除方式繁多，不同部位的肺段切除术难度各异。在胸腔镜肺段切除术的发展历程中，一系列难点逐渐得到解决，如术中肺结节定位，靶段血管、支气管辨认，段间静脉保留，肺段间交界面的分离等，尤其是近几年三维肺支气管血管重建（3D CT bronchography and angiography, 3D-CTBA）的应用，为精准的肺段切除术提供了有力的技术支持^[7]^。

## 1 3D-CTBA的应用

对于拟行较为复杂的肺段切除（如右上叶分段切除、左上叶固有段分段切除、基底段分段切除等）和肺亚段切除，建议术前行3D-CTBA成像，以提高手术的精确性。根据3D-CTBA图像，术前进行模拟肺段切除术，规划手术方案^[7, 8]^。应用3D-CTBA指导胸腔镜肺段切除术的优势在于：

### 1.1 三维显示支气管、肺动脉和肺静脉的解剖结构，判断有无变异、畸形

肺叶支气管、血管的解剖相对简单，而肺段支气管、血管的解剖因人而异、千变万化，因此，肺段切除术较肺叶切除术更为复杂和精细。3D-CTBA可以术前明确、术中指导对于靶段支气管、动脉、段间静脉及段内静脉的精细解剖、准确判断和处理，避免误断肺段支气管和血管而影响保留肺段的通气、换气功能，以期达到个体化的精准解剖性肺段切除。

### 1.2 明确病灶地肺段归属

根据结节与重建的肺段支气管、血管之间的关系，追根索源，判断结节处于哪个肺段，直观、准确地判断结节的肺段归属。

### 1.3 确定肺段切除的手术方案

根据病灶部位、大小、影像学特征及可能的病理性质确定肺段切除方式，行单肺段切除、扩大肺段切除、联合肺段切除、肺亚段切除或者次亚段切除等手术方式，标注需要切断的靶段动静脉、支气管以及需要保留的段间静脉，预定需要分离的段间交界，进行模拟肺段切除。

### 1.4 手术路径的规划

以减少组织损伤、降低手术难度、提高手术精确性为目的，规划最佳手术路径。例如，在拟行右上肺前段、尖段的肺段切除及亚段切除时，可以根据3D-CTBA图像上靶段血管与支气管之间的关系，以肺表面静脉为标记，选择手术最佳切入点。

## 2 手术前结节的定位

对于结节小、位置深或肺磨玻璃样结节（ground glass nodule, GGN），如不采用确切可靠的定位方法，会对寻找病变及判断手术切除范围造成困难。手术前结节定位的优势在于：①术中准确判断小结节在萎陷肺的位置，明确需要切除的肺段，有助于判断是否需要进行扩大的肺段切除术、肺段联合亚段切除术或联合肺段切除术，减少不必要的肺叶切除术。②有助于术中准确判断切缘与小结节的距离，保证足够的手术切缘。③有助于准确发现靶段标本内的小结节，节省术者和病理科医生的时间，缩短等待时间及手术时间^[9, 10]^。

国内外文献报道了多种术前定位的方法，主要包括术中超声定位、术前计算机断层扫描（computed tomography, CT）、引导下注射染色剂、硬化剂、碘对比剂和金属微线圈定位等，各种方法均有其优缺点。我们通常采用术前CT引导下在紧邻病变处注射亚甲蓝及置入导引钢丝（Hookwire），作为胸腔镜手术中定位及病理检查的标记。有专家采用CT引导下置入弹簧圈或注入化学胶定位等方法。

## 3 手术基本操作技术

### 3.1 肺段血管、支气管的处理

肺段血管及支气管较细小，应精细操作，使用的器械包括细的电钩、细的吸引器头、精细剪刀及剥离子等。肺段动、静脉可以沿血管鞘尽可能仔细向远端分离。分离肺段支气管时，可沿着支气管表面，钝、锐性结合向远端分离，亚段支气管较为纤细、壁薄，锐性分离时需要避免损伤。肺段动、静脉细小的分支，可以电凝或超声刀切断，较大的分支可以采用结扎后切断、结扎后超声刀切断，大的分支可使用腔镜用直线切割缝合器切断（缝钉高度2.0 mm-2.5 mm）。不推荐在血管远端使用锁扣夹，以防在切开段间平面时影响直线切割缝合器的使用。肺段支气管的切断采用直线切割缝合器（缝钉高度3.5 mm-4.8 mm），亚段支气管可以结扎后切断。切断支气管及血管后，提起支气管及血管的远端残端，沿支气管及血管鞘膜层次向远端游离，使其远离肺切缘^[11, 12]^。

### 3.2 肺段间交界面的处理

肺段间交界面的确定，通常采用“肺膨胀-萎陷法”：靶段支气管夹闭后张肺，确定靶段支气管辨认正确；靶段支气管切断后张肺，由于存在肺泡间孔，靶段肺组织也膨胀。靶段所在肺叶膨胀后单肺通气，等待其余肺段肺萎陷、约10 min-15 min，靶段肺持续充气，萎陷的肺组织与充气的靶段肺组织之间形成界限，由此判断段间交界面。对于左肺上叶固有段、下肺基底段，确认、切断段支气管后，低潮气量高频通气，待舌段、下叶上段膨胀后使用电凝棒在肺表面电凝作标记，由此判断段间交界。确定段间交界面后，沿标记采用直线切割缝合器（缝钉高度3.8 mm-4.8 mm）切开肺组织。如果分离面明显漏气，可采用生物组织材料及纤维蛋白胶覆盖创面。

其他确定肺段间交界面的方法：①Okada等^[2]^于2007年报道，术中利用超细纤维支气管镜进行选择性靶段支气管高频通气（40 Hz, 2 kg/cm^2^），以确定段间交界。②Misaki等于2009年报道，在靶段动脉切断后静注靛青绿（indocyanine green, ICG; 3.0 mg/kg），在红外线胸腔镜（infrared thoracoscopy）下确定段间交界。③Oh等于2013年报道，靶段支气管切断后，在其远端注入ICG（25 mg溶于50 mL生理盐水），以确定段交界面。

肺段间交界面的分离方法：①电刀及超声刀切开段间交界面。②直线切割缝合器切开段间交界面。③组合使用电刀、超声刀和直线切割缝合器切开段间交界面。

### 3.3 淋巴结采样

对怀疑为恶性病变的患者，手术中应常规做淋巴结采样，应该包括N2及第10、11、12、13组淋巴结，尤其是结节所属的肺段间淋巴结采样不能忽视，送术中快速冰冻病理检查。段间、叶间、肺门、纵隔淋巴结应为阴性。一旦发现有淋巴结转移，对于拟行意向性肺段切除术的患者应该改为肺叶切除术，并行淋巴结清扫。

### 3.4 楔形切除与肺段切除

位于肺脏层胸膜下的结节，先行楔形切除术，术中快速冰冻病理明确为恶性后行肺段切除术；对于病变性质不明、位置深、无法行楔形切除术者，直接行肺段切除术。

## 4 术中注意事项

### 4.1 肺段血管、支气管的辨认和处理

肺段支气管及血管较细小且变异较多，需要沿支气管及血管根部尽可能向远端仔细分离、辨认。肺段动脉与支气管伴行，术中可以相互参照。肺段静脉的主要分支走行于肺段之间，称之为段间静脉（intersegmental vein），其沿途的分支为走行于肺段内及亚段之间的静脉（段内静脉，intrasegmental vein），在处理肺段静脉时，尽可能只切断段内静脉、保留走行于靶段与邻近肺段之间的段间静脉。在无法确认段间静脉时，尽可能将靶段的各段间静脉向远端分离，在确定段间交界面后连同肺实质一并切断，避免损伤和误断。靶段支气管辨认困难时，需要术中进行纤维支气管镜检查，并借助其光源定位靶段支气管。

### 4.2 肺段间交界面的解剖性分离

术中切断靶段动脉、支气管后，膨胀萎陷法确定脏层胸膜的段间交界线，一般等待10 min-15 min左右，均可显示明显的段间交界面。提起靶段支气管、血管远侧残端，沿段间静脉按照膨胀萎陷平面由肺门向肋面用电勾仔细分离段间交界面。沿途切断由段间静脉发出走向靶段内的段内静脉，保留相邻段的段内静脉，解剖性分离段间段间交界。当分离至所剩肋面的靶段段间肺组织厚度在1 cm-2 cm（膨胀状态）时，使用腔镜切割缝合器切割分离剩余的段间肺组织。

### 4.3 淋巴结的处理

对于早期非小细胞肺癌（non-small cell lung cancer, NSCLC）患者，可以进行严格、精准的术前淋巴结的临床分期，包括CT、正电子发射计算机断层显像（positron emission tomography/ computer tomography, PET/CT）、纵隔镜、气管镜超声引导针吸活检（endobronchial ultrasound needle aspiration, EBUS-NA）和/或食管镜超声引导针吸活检（endoesophageal ultrasound needle aspiration, EUS-NA）等检查。但是，由于可能存在淋巴结的微转移和跳跃转移，以及各项检查均有其局限性，且敏感性、特异性均不能达到100%，尤其是淋巴结 < 1 cm时，所以，部分cT1N0术后升级为pT1N1-2。因此，NCCN指南总是提出术中应该常规进行纵隔（N2）、肺门（第10组）、叶间（第11组）、肺内（第12组）、段间（第13组）淋巴结采样，并送快速冰冻病理检查。Ikeda等推荐，术中对第12、13组及肺叶特异性N2（最大及第二大的淋巴结）切除送检。如果发现有淋巴结转移，除非是需要进行妥协性肺段切除术，否则应该中转改为肺叶切除术。

### 4.4 切缘

对于NSCLC患者，需要保证切缘≥2 cm或切缘/肿瘤直径≥1 cm。如果切缘不能达到要求，则应该进行扩大的肺段切除或联合肺段切除，必要时改为肺叶切除术。

### 4.5 避免保留肺段支气管、血管的损伤以及靶段支气管、血管的残留

靶段血管、支气管切断后，需要提起其远侧残端，尽可能向远侧剥离，使其远离肺门结构，并保留段间静脉，以免切开段间平面时损伤需要保留肺段的支气管、血管。沿着段间平面切开肺段组织、尤其在接近肺门时，使用直线切割缝合器要特别小心，需要将靶段支气管与血管的远侧残端推开，使之位于直线切割缝合器的靶段一侧，以防残留。

### 4.6 注意保留的肺段内有无活动性出血

由于肺段之间没有肺裂，段间交界面的切断部分是由直线切割缝合器完成，在切断段间肺组织时可能会损伤段间静脉，引起保留肺段肺组织出血或血肿，术中需要麻醉师吸痰并确定气道内有无活动性出血。如果有活动性出血，需要用4-0 prolene线连续缝合加强切缘，必要时改为肺叶切除术。

### 4.7 避免靶段肺组织残留过多

靶段肺组织残留过多，表现为段间交界面切缘附近有局限性肺组织持续不张或过度充气，应予切除。

### 4.8 预防肺扭转

靶段切除后，需要注意预防保留肺段的扭转，如左上叶固有段切除后保留的舌段、下叶基底段切除后保留的上段。手术结束时张肺，将该保留肺段保持在自然位置；也可以采用缝合的方法或使用直线切割缝合器，将其固定于邻近的肺叶。

## 5 手术后常见并发症和处理

### 5.1 咯血

主要原因为：①损伤段间静脉，分离段间平面时容易发生。②误断保留肺段的静脉，导致保留肺段血液回流受阻，称为“静脉梗死”。精准的解剖性肺段切除术很少引起咯血并发症，但开展初期由于经验不足，或血管变异时辨认不清，误断保留肺段的静脉，或者段间平面辨认不清而损伤段间静脉。

处理方法：①少量咯血，表现为痰中带血，可口服卡络磺钠、云南白药等，或用蛇毒类止血药，一般3天-7天可治愈。②大量咯血，止血药无效时，需要再次手术切除保留的肺段。

### 5.2 漏气

主要原因为：①解剖、分离肺段血管、支气管和淋巴结时损伤脏层胸膜。②使用电刀切开段间肺组织时容易发生漏气。③使用直线切割缝合器切开段间肺组织，切割线上缝合钉处漏气，肺气肿患者多见。④损伤支气管。

如果严格按照肺段间交界分离，很少发生术中及术后漏气。术中可以使用修补材料如奈维覆盖创面，并使用生物蛋白胶喷洒在创面，如发现支气管损伤，必须缝合修补。术后处理方法：①少量漏气可延长胸管引流时间，在数日内自愈。②也可胸腔内注射粘连剂，如高糖、凝血酶、红霉素等，行胸膜固定。③长期大量的漏气，要注意有无支气管胸膜瘘的存在，处理同支气管胸膜瘘。

## 6 胸腔镜解剖性肺段切除术的进展

解剖性肺段切除术的精髓为靶肺段的解剖性切除，包括靶段动脉、静脉、支气管的分别切断，保留段间静脉，段间交界的解剖性分离。解剖性切除的目的是为了完整切除靶段，并且最大程度的使保留肺段发挥功能，这充分满足肺癌手术的要求：最大限度地切除病灶，最大限度地保留肺组织，同样也是符合肺段切除术的初始目的：保护术后肺功能。

目前通用的解剖性肺段切除术的概念仅局限于靶段支气管、动脉的解剖性切除^[13, 14]^，对段间静脉的保留和肺段间交界的解剖性分离并无指导意义。肺段与肺叶的解剖关系：若干个不规则锥形结构的肺段拼接成一个肺叶，锥尖为肺段支气管，段间静脉行走于肺段间。本中心探索总结并命名一种按照锥形结构原理自肺叶中分离出肺段的方法-“锥式肺段切除术”，以达到完全解剖性肺段切除。通过前期研究发现锥式肺段切除术安全可行，可精准切断靶段支气管、动脉、段内静脉，保留段间静脉，解剖性分离段间交界，达到精准的解剖性肺段切除，可确保肺癌结节的安全切缘宽度、减少切缘肺组织的重叠压榨、保持剩余肺段的几何形状。

## 7 总结

胸腔镜肺段切除术安全可行，技术逐渐完善不断进展，解剖性切除是主要发展方向，在保证安全切缘的前提下尽可能多的保留肺组织，并且使保留肺段充分发挥功能，3D-CTBA在精准解剖性肺段切除术中发挥重要作用。
